# Embolized Watchman Removal With Modified Hangman Technique

**DOI:** 10.1016/j.jscai.2024.102072

**Published:** 2024-05-18

**Authors:** Jonathan Charles, Premsai Kumar, Summer Decker, Jonathan Ford, Glenn Hoots, Kamal Massis, Brandon Pagan, Jamil Shaikh, Clifford Davis

**Affiliations:** USF Health Morsani College of Medicine, Tampa, Florida

**Keywords:** embolization, left atrial appendage occlusion, modified hangman, Watchman

## Abstract

In this case report, we explore a novel technique to remove an embolized Watchman device (Boston Scientific) into the thoracic aorta endovascularly. The technique involves a wire + snare combination that is threaded through the metal struts of the Watchman. This combination technique along with the threading provides increased stability during removal and decreases the risk of the Watchman slipping from the devices and causing further embolization. Further work is required to elucidate the efficacy of this technique in other scenarios.

## Introduction

In this work, we showcase a previously undescribed method of endovascular removal of an embolized left atrial appendage occlusion device (LAAD) utilizing a combination of snare and wire, herein referred to as the “modified hangman” technique.

## Methods

On CT examination, an 82-year-old man with uncontrolled atrial fibrillation demonstrated embolization of his previously implanted LAAD distally into his abdominal aorta. The patient was likely not a candidate for rhythm control and long-term oral anticoagulation; therefore, the decision to implant the LAAD was made. The patient was transferred to Tampa General Hospital for further treatment. Interventional radiology and vascular surgery were consulted for endovascular removal of the device.

Under fluoroscopy, a Bentson wire (Boston Scientific) was advanced through the right femoral access and a stiff Lunderquist (Cook Medical) wire through the left femoral access. A 20F DrySeal sheath (GORE) was placed in the right and an 18F dry seal sheath in the left.

The Bentson was exchanged for a 260-cm angled GLIDEWIRE (Terumo), which was advanced proximal to the device. Through the right, a 6F loop snare was introduced and advanced proximal to the device. The end of the glidewire was snared. Consequently, the wire and snare combination provided a secure, closed-loop grip on the device.

**Figure 1:**
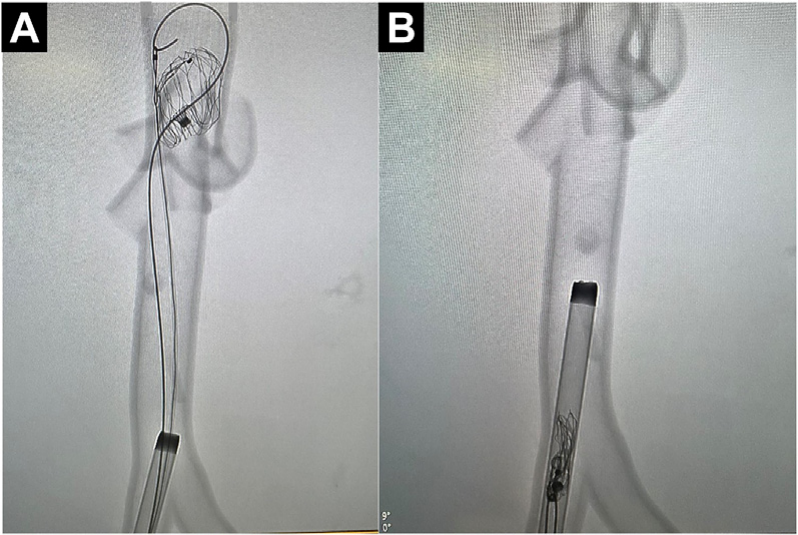
**Fluoroscopicvisualization of the embolized Watchman device and wire + snare combination.** (**A**) The snare is securely closed around the wire tip. (**B**) The entire wire + snare + Watchman assembly is removed through the sheath.

The assembly including the Watchman device was retracted under tension through the lumen of the 18F sheath and removed. Vigorous aspiration with 60-cc syringes was performed concomitantly through bilateral sheaths with the removal to prevent possible distal thromboembolism.

## Discussion

The hangman technique, although a well-described method for removal of an inferior vena cava filter, modified for removal of an embolized LAAD within the aorta, has not been previously described to our knowledge.

**Figure 2:**
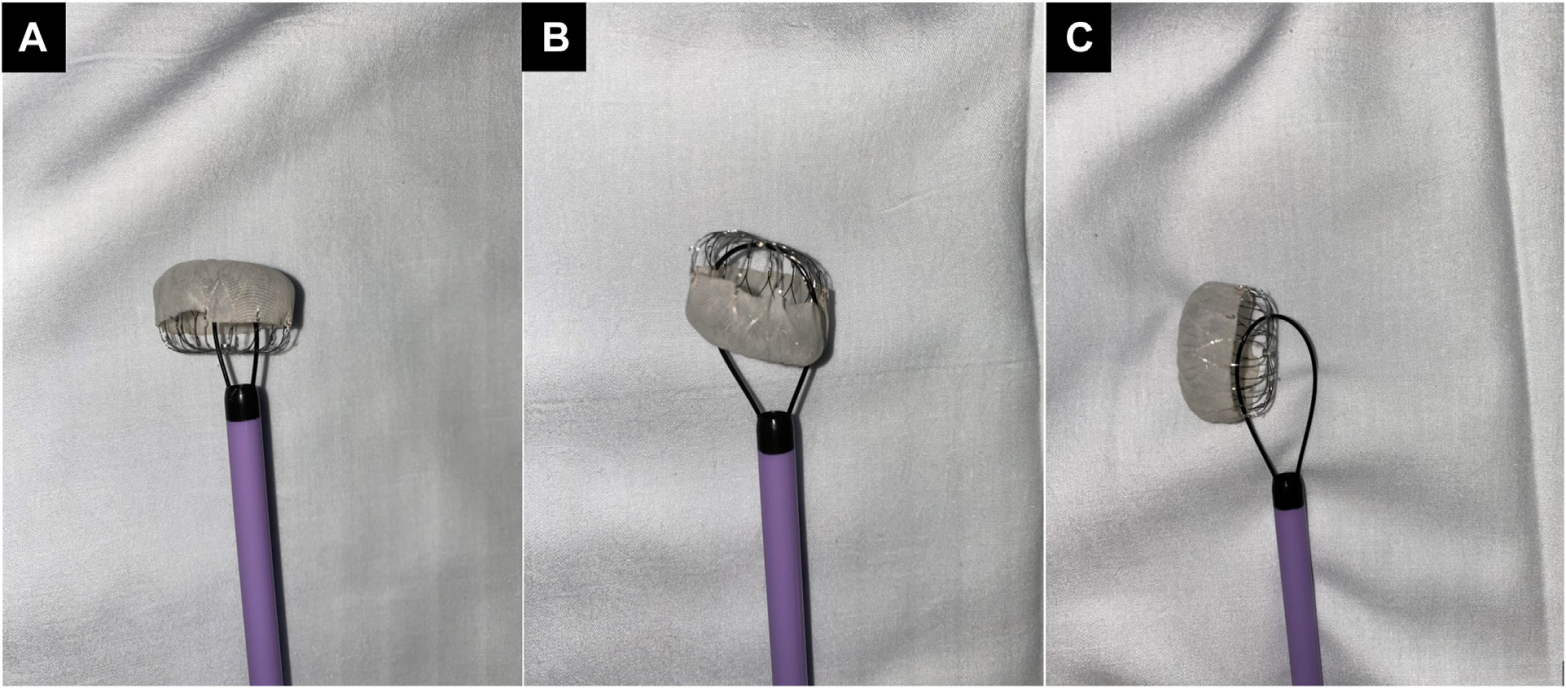
**The 3 possible orientations of the embolized Watchman device with appropriate wire + snare setups.** (**A**) Top of the device facing cephalad. (**B**) Top of the device facing caudad. (**C**) Top of the device levo/dextroverted.

Three orientations of the embolized LAAD are possible (Figure 2). This device was oriented sagittally with the steps for removal listed in the Methods. When the device is oriented cephalad or caudad, the wire enters around the device struts, buckles against the inner surface, and curves outwards proximally from the device, forming a “U.” The snare will then capture the free end of the wire, anchor it, and the snare + wire + LAAD combination will be retracted through the 18F sheath. If the wire is unable to be directed into the LAAD’s open basket portion, a reverse curve catheter can be used to direct the wire through the struts. Ex vivo testing with LAAD and sheaths found that the smallest sheath the device would comfortably fit through would be 16F.

**Figure 3:**
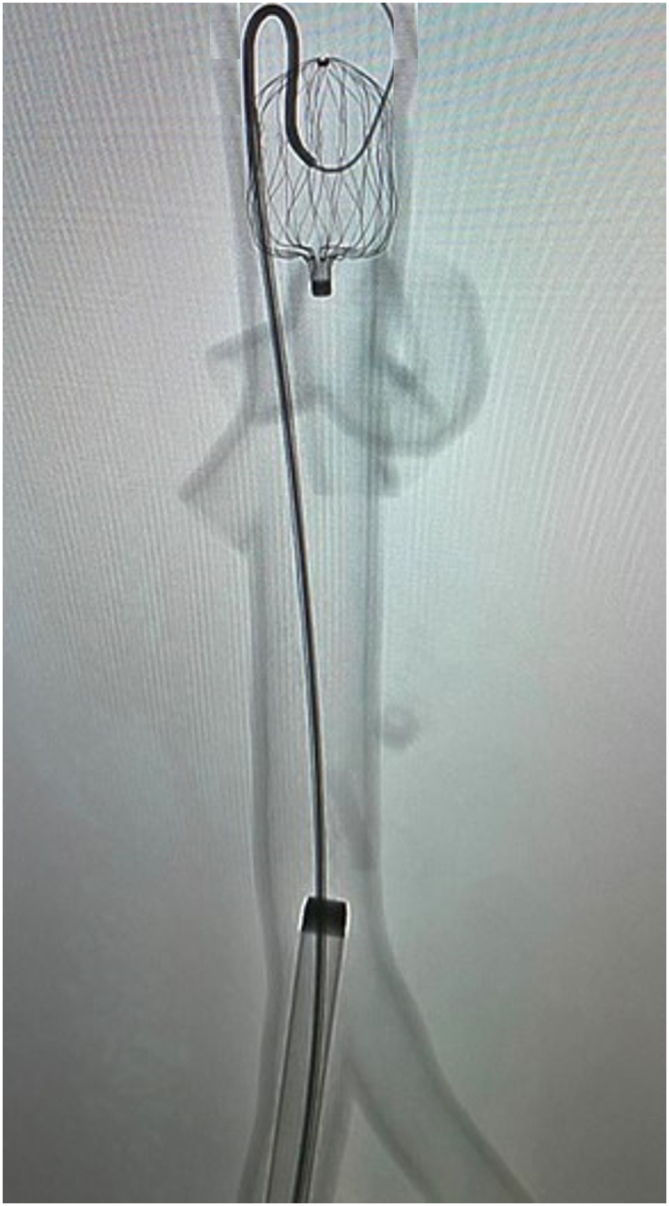
**The reverse curve catheter being utilized to help “point” the wire in the desired direction into the Watchman device**.

A retrograde aortic approach through the common femoral artery is best for embolizations into the thoracic and abdominal aorta. Contrarily, a transseptal approach is preferred when the retrieval is planned from the left atrium or pulmonary veins.

### Limitations

Microembolism during endovascular manipulation is a concern. Cerebral embolization protection devices have shown variation in efficacy of embolic protection.^1^ In high-risk cases, these devices can be considered. Additionally, a filter could be implanted into the aorta distally or proximally to prevent embolization into renal, femoral, or mesenteric arteries, a technique previously described by Turagam et al.^2^ Further limitations include the case series nature of this reporting.
